# Current Strategies for Cancer Cell-Derived Extracellular Vesicles for Cancer Therapy

**DOI:** 10.3389/fonc.2021.758884

**Published:** 2021-11-05

**Authors:** Weijian Lin, Xing-Dong Cai

**Affiliations:** ^1^ Department of Oncology, The First Affiliated Hospital of Jinan University, Guangzhou, China; ^2^ Department of Respiratory, The First Affiliated Hospital of Jinan University, Guangzhou, China

**Keywords:** cancer cell-derived extracellular vesicles, cancer therapy, nanomedicine, drug delivery, advanced materials

## Abstract

Cancer cell-derived extracellular vesicles (CEVs), a novel type of therapeutic agent in cancer treatment, can be prepared from the autocrine secretion of various cancer cells, the direct extraction of cancer cells and the combination of cancer cell-derived membranes with advanced materials. With various bioactive molecules, exosomes are produced by cells for intercellular communication. Although cancer cell-derived exosomes are known to inhibit tumor apoptosis and promote the progression of cancer, researchers have developed various innovative strategies to prepare anti-tumor vesicles from cancer cells. With current strategies for anti-tumor vesicles, four different kinds of CEVs are classified including irradiated CEVs, advanced materials combined CEVs, chemotherapeutic drugs loaded CEVs and genetically engineered CEVs. In this way, CEVs can not only be the carriers for anti-tumor drugs to the target tumor area but also act as immune-active agents. Problems raised in the strategies mainly concerned with the preparation, efficacy and application. In this review, we classified and summarized the current strategies for utilizing the anti-tumor potential of CEVs. Additionally, the challenges and the prospects of this novel agent have been discussed.

## Introduction

**Figure 1:**
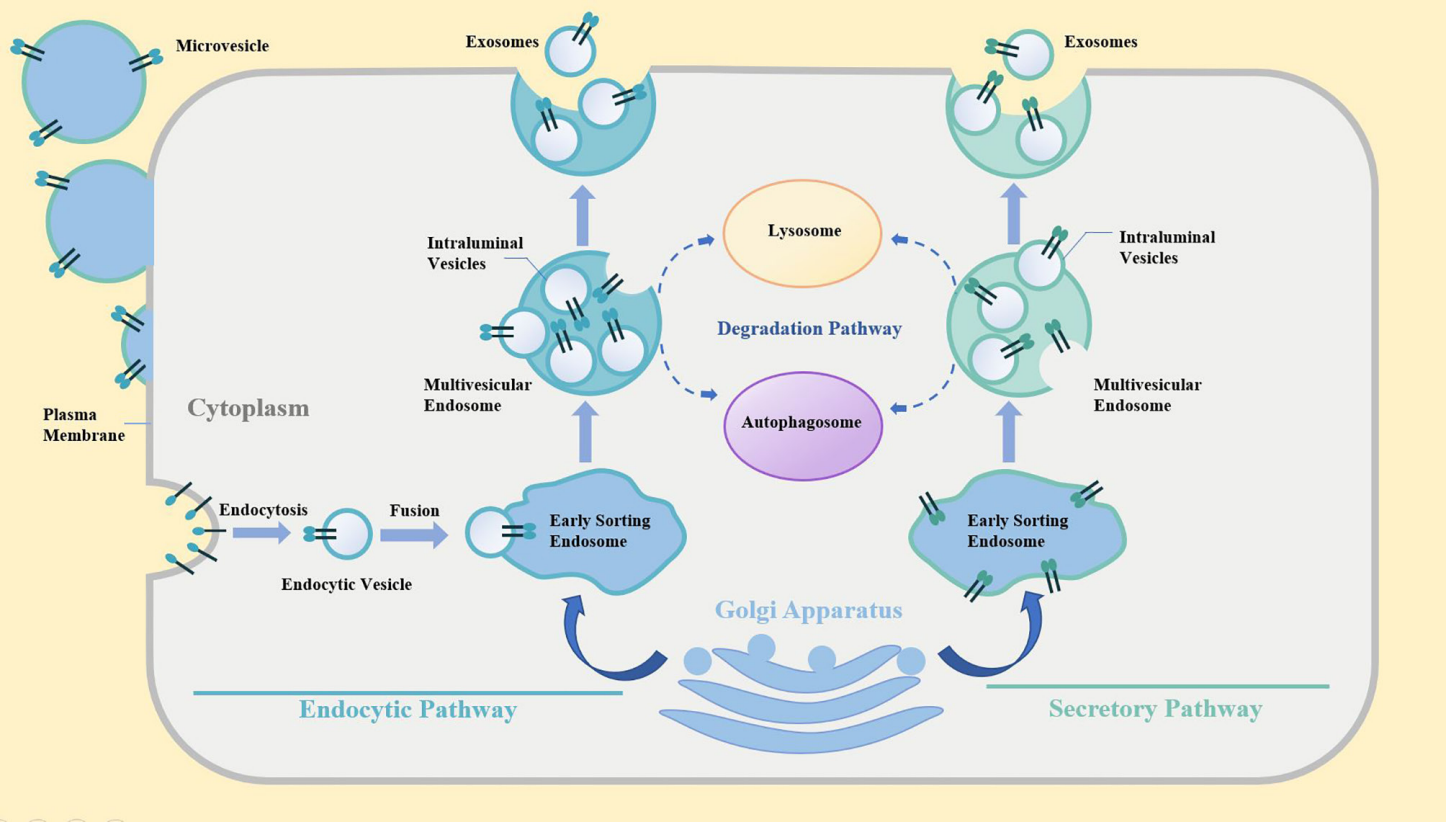
The biogenesis of exosomes.

Extracellular vesicles (EVs) are phospholipid bilayer membrane-coated vesicles that are generated by cells for intercellular communication (1, 2). EVs can be classified into exosomes, microvesicles and apoptotic bodies (3, 4). The exosomes biogenesis was showed in **Figure 1**. The classification of EVs is mainly based on their biogenesis. Ranging from 100 nm to 5 μm, apoptotic bodies were generated and secreted by the cells undergoing apoptosis with outward blebbing of the plasma membrane (5, 6). With a diameter from 50 nm to 1 μm, microvesicles generated by budding from cellular membranes after activation, shear, or physical stress (7). Exosomes are defined as the vesicles with a size of 30-150 nm, and are released from cells undergoing fusion of an endocytic compartment multivesicular body with the plasma membrane (8). EVs are inherently loaded with cargoes, including various bioactive molecules, such as nucleic acids, proteins, and lipids for cell-to-cell signaling (9). Therefore, the short/long distant exchange of bioactive factors in cells is closely associated with the transfer of EVs in health and disease. Regarding the EVs derived from cancer cells, EVs are naturally the key players in tumor progression, tumor metastasis, immunosuppression, and drug resistance (10–15). The presence of pernicious products in vesicles limits, to a great extent, the exploration of the therapeutic value in Cancer cell-derived extracellular vesicles (CEVs) but still fail to cover the latent anti-tumor capacity of vesicles.

In the past decade, there has been extensive research done on CEVs, which has facilitated their use in the development of novel therapeutic agents for cancer therapy. Several *in vitro*, *in vivo*, and clinical trials have been conducted using CEVs-based strategies to obtain CEVs with effective anti-tumor activity. One of the primary advantages of CEVs includes their high targetability to the tumor. Additionally, they are known to preferentially target their homologous tumor cells (16–20). The specific accumulation of CEVs in the tumor area implies that CEVs could be used for drug delivery for potential targeted therapy. Thus, recent studies investigated the effects of drug-loaded CEVs and found excellent effectiveness against cancer (21–26). Additionally, oncolytic viruses can also be packed into CEVs to protect them from neutralizing antibodies in the serum, allowing them to function as oncolytic agents (27). The parent cancer cells, which secrete CEVs, heavily determine the constituents, determining the essential quality of CEVs. Studies have revealed genetically engineered cancer cells, which generate genetically engineered CEVs, resulting in efficient anti-tumor immunity and improved anti-tumor efficacy (28–33). Additionally, cancer cells were also irradiated to prepare CEVs to enhance the safety and anti-tumor efficacy of CEVs. With a potential role in exosome-mediated bystander effects (34), these irradiated CEVs could inhibit the progression of the tumor (25, 35).

Several studies have indicated that EVs from cancer can promote tumorigenesis and metastasis; thus, the EVs derived from immune cells, such as dendritic cells, natural killer cells, and CAR-T cells, have been used for cancer therapy (36–40). The in-depth analysis of CEVs, as well as the exploration of therapeutic EVs of immune cells, have provided indications for the role of CEVs in cancer therapy. In this review, we provide a comprehensive overview of the current strategies, challenges, and prospects for the use of CEVs in cancer treatment.

## Irradiated Cancer Cells-Derived Extracellular Vesicles

**Figure 2:**
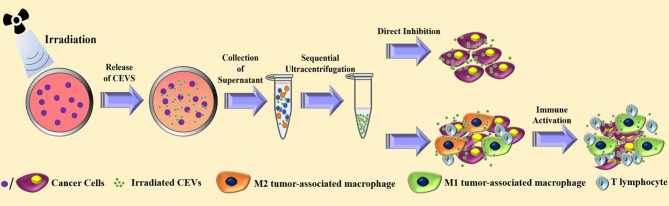
The current strategies for the preparation and anti-tumor effect of irradiated cancer cells-derived extracellular vesicles.

Previous studies have shown that radiotherapy, one of the primary treatments in cancer therapy, can cause a radiation-induced bystander effect (RIBE), which is a unique reaction triggered by irradiated cells or tissues, leading to the induction of apoptosis in unexposed cells (41–45). Similar to RIBE, the ionizing radiation-exposed cancer cell vaccines were tested, and their efficacy was confirmed in clinical trials (46–48). Researchers showed that the underlying mechanism of irradiated tumor cell vaccine in cancer therapy involved the activation of dendritic cells and T cells (49–51). Hence, radiation exposure was used in the preparation of CEVs. **Figure 2** illustrates the mechanism involved in the preparation of irradiated CEVs.

In a recent study on irradiated CEVs, the researchers irradiated the cancer cells with a single dose of 20 Gy by 6-MV X-rays to generate EVs (35). The results showed the irradiated CEVs could induce the ferroptosis of cancer cells. Additionally, the treatment with irradiated CEVs in the mouse model induced the transformation of M2 tumor-associated macrophages into M1 tumor-associated macrophages. For CEVs loaded with chemotherapeutic agents, the process of irradiation was able to improve the efficacy in the cancer treatment (51). Another study showed that compared with the PBS group, CEVs of A549 lung cancer promoted the progression of cancer and malignant pleural effusion, while the irradiated CEVs group showed no significant change in the cancer progression (25). Additionally, the irradiated and drug-loaded CEVs of A549 lung cancer inhibited the progression of cancer and malignant pleural effusion in the clinical tests (25, 52).

Although the anti-tumor efficacy of irradiated CEVs was triggered by the promotion of tumor antigen (53), the detailed mechanism of the RIBE is unclear and the presentation of tumor antigen may associate with the therapeutic Dexosomes (one kind of exosomes secreted form Dendritic cells) (54). Further studies are required to explore the underlying mechanism of irradiated CEVs against cancer.

## Advanced Materials Combined Cancer Cells-Derived Extracellular Vesicles

**Figure 3:**
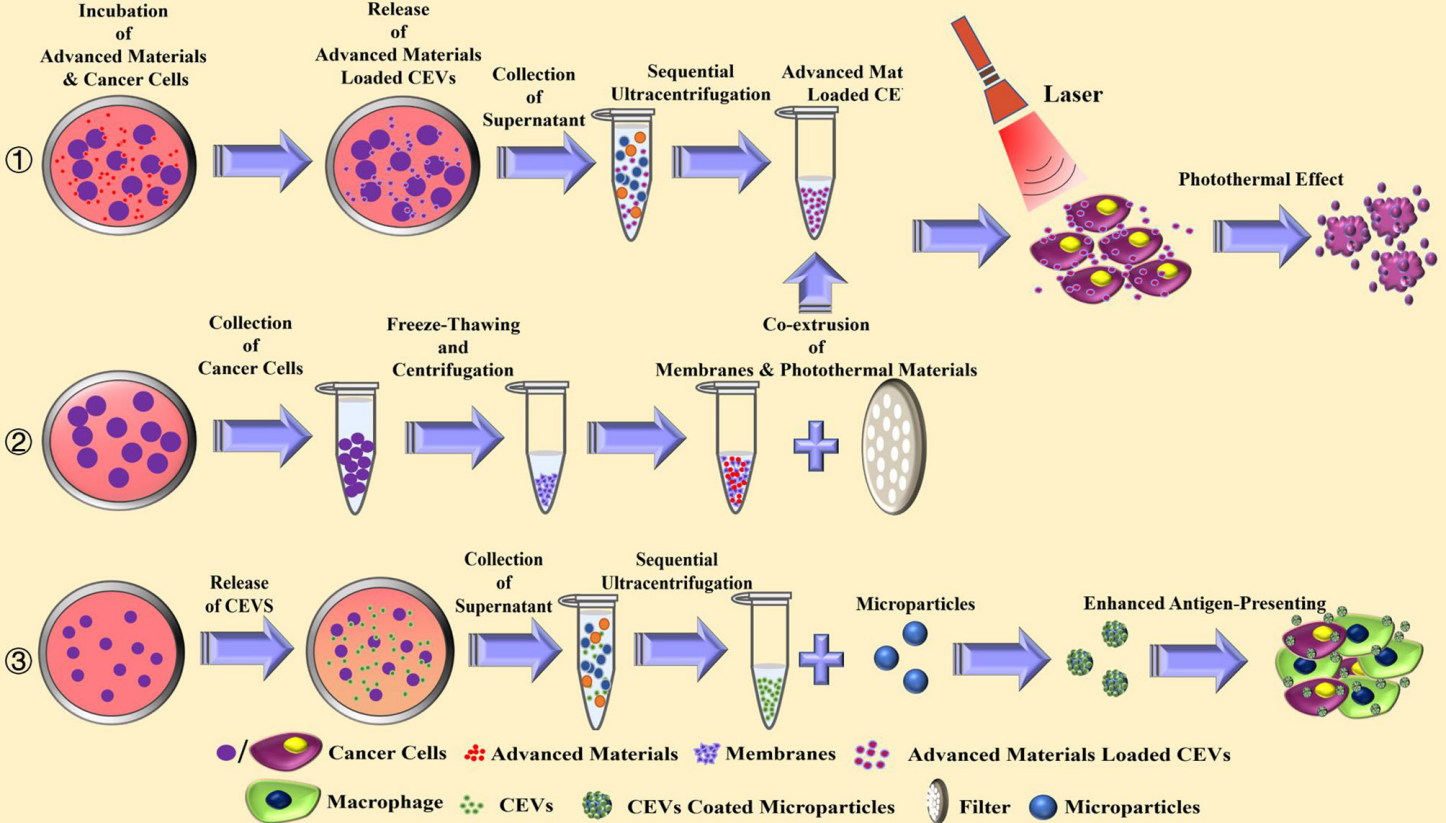
The current strategies for the preparation and anti-tumor effect of advanced materials combined cancer cells-derived extracellular vesicles.

There has been an extensive increase in the use of advanced materials as biomimetic carriers in the field of cancer therapy. Nanoparticles serve as stable carriers to protect antigens from rapid clearance and degradation in the serum, achieving a stronger immune response (55–57). In a study, PLGA microspheres were coated with CEVs, and the results showed an increase in phagocytosis in macrophages and dendritic cells (58). Consequently, treatment with coated CEVs caused an enhanced cytokine release in antigen-presenting cells. **Figure 3** illustrates the mechanism involved in the coating of CEVs with advanced materials.

When therapeutic materials, such as photothermal materials and cytotoxic agents, are combined, CEVs serve as carriers to accumulate in tumor cells leading to cytotoxicity. The pristine zinc oxide nanocrystals enclosed with CEVs could be engulfed by cancer cells, exhibiting toxicity (59). Also, photothermal therapy was designed to combine with CEVs, based on its direct cytotoxic effect and ability to increase the susceptibility of cancer cells to chemotherapy. CEVs were loaded with photothermal Bi_2_Se_3_ and chemotherapeutic doxorubicin hydrochloride (21). With good biosafety and feasibility, the CEVs could disperse well in the tumor area, exhibiting photothermal effect.

Most studies on CEVs for advanced materials combined with biomimetic EVs were performed using membranes of cancer cells. The design of cancer cell membrane-coated nanoparticles is based on the homologous target for targeted drug delivery (18, 60–63). The membrane is extracted from cancer cells mostly *via* freeze-thawing and centrifugation. The photothermal materials loaded-nanoparticles with cancer cell membrane were assembled by the co-extrusion of their mixture to generate the biomimetic CEVs (18, 63). The membrane from hybrid cells of cancer and dendritic cells were extracted and combined with photothermal nanoparticles against cancer to enhance the antigen-presenting function of biomimetic EVs, resulting in a promising anti-tumor effect (16).

## Chemotherapeutic Drugs Loaded Cancer Cells-Derived Vesicles

**Figure 4:**
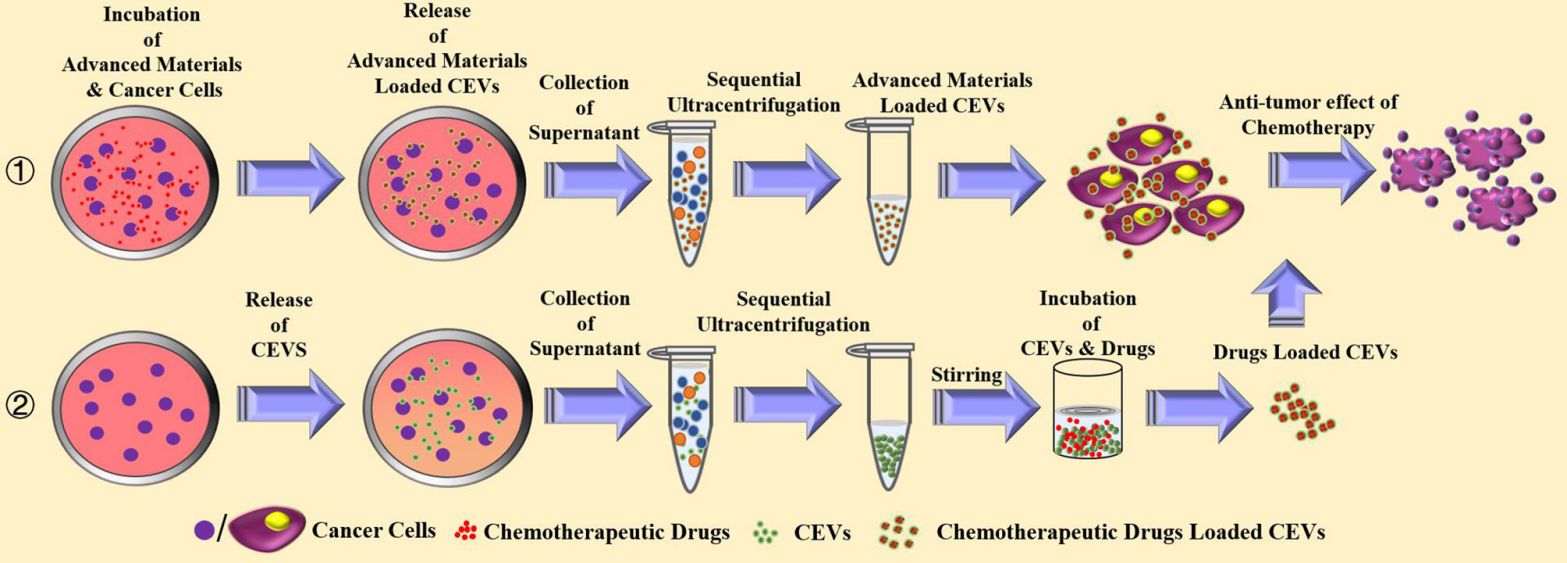
The current strategies for the preparation and anti-tumor effect of chemotherapeutic drugs loaded cancer cells-derived extracellular vesicles.

CEVs possess the properties of homologous tumor targeting and favorable compatibility for drugs. **Figure 4** illustrates the mechanism involved in the loading of chemotherapeutic drugs into CEVs. The drug-loaded CEVs are mostly prepared by the co-incubation of drugs with cancer cells, resulting in the phagocytosis of drugs. As the EVs are processed and generated, CEVs are released and packed with chemotherapeutic drugs in the supernatant of cancer cells (21–23, 25, 51, 64). In other studies, the drug-loaded CEVs were mainly prepared in two steps: the first step involved the isolation of CEVs, and the second step involved drug loading (19, 26, 65–67). The first step of isolation was conducted mainly *via* the traditional ultracentrifugation. In a study of doxorubicin-loaded cell-derived nanovesicles for the generation of CEVs (66), U937 lymphoma cells were sequentially extruded through filters using 10 and 8 μm filter membranes, followed by purification using Sephadex G50 size-exclusion column. After collecting CEVs, the second step of drug loading was performed *via* the direct incubation of CEVs with chemotherapeutic agents at 37°C (26, 66) or 22°C (67). However, compared with incubation at room temperature, a better efficiency of drug loading and release was observed at 37°C (66). Additionally, the co-extrusion of the mixture of chemotherapeutic drugs and CEVs also exhibited efficient drug loading and anti-cancer activity (19). The sonication of the mixture of catalase and EVs of macrophages showed the highest loading efficiency and good releasing efficiency, among other methods of direct incubation, freeze-thawing, and co-extrusion (68).

Additionally, oncolytic virotherapy was also used in combination with CEVs against cancer. Oncolytic adenovirus infected- A549 lung cancer cells could release EVs containing bioactive adenovirus particles (27). The CEVs can protect adenovirus from the clearance in the serum, resulting in improved anti-cancer efficacy. Although viruses combined with CEVs could exhibit therapeutic efficiency, upregulated levels of CLTA-4 and PD-1 were detected after the administration of the virus for the cancer therapy (69–71), and the PD-L1 of cancer EVs also promoted tumorigenesis (72). Thus, further studies are required to design combined therapy, including immunotherapeutic drugs with EVs, by constructing CEVs with immune checkpoint inhibitors.

In clinical experiments, chemotherapeutic drugs-loaded CEVs achieved promising outcomes in lung cancer patients with malignant pleural effusion (MPE). Cisplatin-loaded CEVs were prepared from the supernatant of the mixture of A549 cells and Cisplatin (52). The injections of cisplatin-loaded CEVs were administered intrathoracically four times, and the results exhibited a 95% reduction in tumor cells in the MPE. In another clinical study with methotrexate-loaded CEVs (25), autologous tumor cells were prepared from patients’ MPE and incubated with chemotherapeutic methotrexate after ultraviolet irradiation. The methotrexate-loaded CEVs were collected by ultracentrifugation. Autologous CEVs were administered intrapleurally six times. This therapy could effectively stimulate CD4+ T cells and showed a good safety profile of CEVs, exhibiting\ promising reduction in tumor cells and CD163+ macrophages.

## Genetically Engineered Cancer Cells-Derived Extracellular Vesicles

**Figure 5:**
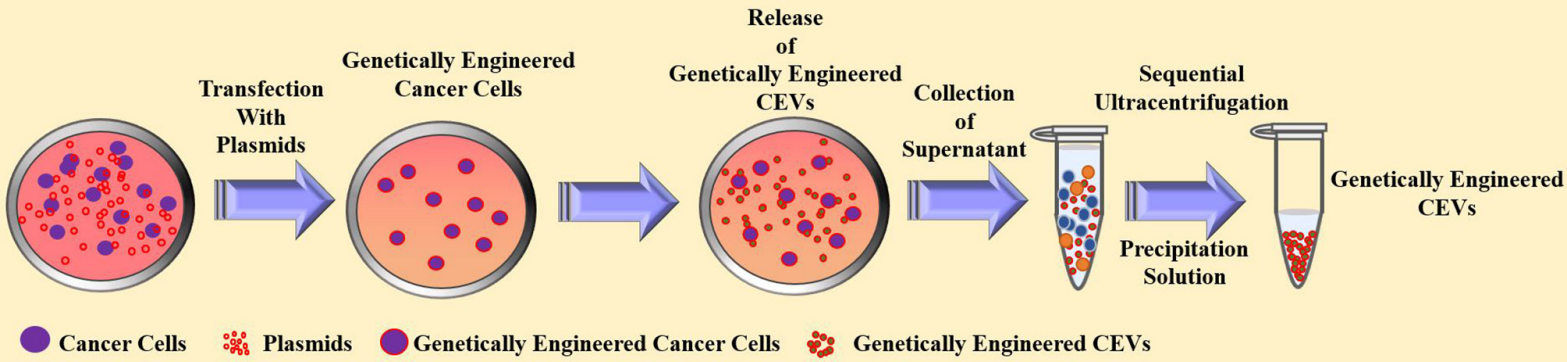
The current strategies for the preparation and anti-tumor effect of genetically engineered cancer cells-derived extracellular vesicles.

CEVs are the final products generated and prepared from cancer cells. Therefore, the construction of a genetically modified CEVs requires a genetically engineered cancer cell line. Thus, various design strategies were designed by transfecting therapeutic genes into cancer cells to alter the inherent characteristics of CEVs for cancer therapy (28–31, 33). **Figure 5** illustrates the design strategies for genetically engineered CEVs.

The miRNA content of the exosomes is also known to promote the progression of cancer (73), such as miRNA-15b (74) and miRNA-21 (75), while exosomal miRNA-22 (76), miRNA-142 (77), and miRNA-155 (31, 78) have been shown to exert therapeutic effects against cancer. A study showed that after transfecting DNA vectors expressing miRNA-155 and miRNA-125b2 into pancreatic cancer cells, the altered CEVs had upregulated miRNA levels, as detected by the ExoQuick-TC exosome precipitation solution (31). The results showed that the genetically engineered CEVs promoted the reprogramming of macrophages to the M1 phenotype. Additionally, miRNA-21 is known to enhance chemotherapy resistance; thus, a combination of silencing such miRNA and CEVs showed improved chemotherapy sensitivity. Also, treatment with anti-miRNA-21-loaded CEVs and doxorubicin arrested the growth of cancer cells (30).

Also, CEVs are known to naturally possess the proteins that present in the corresponding cancer cells, such as immunosuppressive PD-L1 (72) and prostate-specific antigen (PSA) (79). In a study, B16BL6 cells were transfected with the plasmid expressing streptavidin and lactadherin, followed by ultracentrifugation to isolate the CEVs to endow them with increased capacity of tumor antigen presentation (33). In another study, the engineered EVs derived from Expi293 cells expressing anti-CD3 and anti-HER2 antibodies exhibited a potent anti-tumor effect for targeted immunotherapy. Additionally, the therapeutic proteins in CEVs could be overexpressed *via* transfection to boost the therapeutic efficacy of inherent proteins in CEVs. An efficient induction of anti-tumor immunity was observed after treatment with CEVs that overexpressed Rab27a (28), which is known to serve as an important regulator of exosomes (80) and antigen presenters (81). It is noteworthy that the genetically engineered CEVs which had experienced irradiation could serve as a much more effective medicine against cancer (82).

## Challenges and Future of Cancer Cells-Derived Extracellular Vesicles

### Strategy for Preparation

Although gradient ultracentrifugation provides excellent purity for the preparation of CEVs, sequential ultracentrifugation is more commonly used as it is more convenient and provides a larger volume. The polymer precipitation technique is easy to perform and efficient; however, it has a complicated clean-up process. Additionally, novel strategies, such as microfluidics-based techniques and immunoaffinity capture, are also suitable for the isolation of CEVs; however, they are expensive.

Thus, new techniques are required for the innovative generation of self-designed CEVs for further analysis of the anti-tumor potential of CEVs. A sequential extrusion strategy was used for the artificial synthesis of CEVs. CEVs were prepared after extrusion through 10 and 8 μm membrane filters (66). Additionally, the process of freeze-thawing of cancer cells and co-extrusion with cancer cells and nanoparticles were used to generate CEVs with the cancer cell membrane and advanced nanoparticles (18, 63).

### Strategy for Efficacy

Before CEVs reach the tumor area, the number of intact EVs determine the therapeutic efficacy of CEVs. The efficacy of CEVs is largely dependent on the stable construction and good targetability after preparation and preservation.

Since the EVs derived from cancer cells can be important promotors of tumor progression, tumor metastasis, immunosuppression, and drug resistance (10–14, 83), irradiation is used for the preparation of therapeutic CEVs to reduce vicious side effects (25, 35, 49, 51, 53, 84). Cancer cells are prone to apoptosis after irradiation, which positively influences the construction of cancer cells membrane and the membrane of CEVs, leading to a damaged stability of CEVs. Besides, the resistance against radiotherapy maybe induced *via* the communication of irradiated exosomes (85). Additionally, the pH of the reserve solution plays a controversial role in the preservation of CEVs (86, 87), although EVs retain their quality for 16 days at -80°C (88). Studies showed the core-shell structure to construct biomimetic EVs (60, 61) and the hydrogel encapsulated exosomes (89) can result in enhanced stability.

Although the cargo carried by the EVs play a vital role in exerting a suitable therapeutic effect, their efficacy is also determined by their targetability and the effective functioning in the tumor. For the efficacious transport of CEVs to the tumor, the targetability of CEVs could be enhanced *via* the modification of tumor-targeted-aptamers (62) and peptides (84) on EVs. Along with specific accumulation, the absorption and the anti-tumor activity in the tumor area are also crucial, especially for drug-loaded CEVs. After homologous tumor targeting, the pH-sensitive fusogenic peptides-modified CEVs exerted a membrane-lytic activity in the tumor microenvironment to enhance the effective delivery and release of the cargoes (33).

### Strategy for Application

CD47, on EVs in cancer patients, carries a “don’t eat me” signal (90); thus, CEVs might overexpress CD47 in their parent cancer cells (91–93) to escape the phagocytosis and rapid clearance of macrophages in the serum. Thus, several studies intravenously administered CEVs (19, 21, 23, 26, 51). However, numerous phospholipid bilayer membrane-coated CEVs were also degraded and accumulated in the lung and liver (19, 21). Therefore, an intrapleural administration was adopted to treat lung cancer patients with MPE to avoid unnecessary clearance (25, 52).

In the bright side, the newest researches showed the CEVs were able to be used for the trace of the early stage of neoplastic transformation (94) as well as the intracranial tumor may also can apply the CEVs because they can penetrate the blood-brain barrier (BBB) and have an extraordinarily high brain delivery efficiency (95).

In the clinical experiments, the drug resistance of tumor-repopulating cells can be reversed after the application of CEVs (52). Besides, the autologous chemotherapeutic drugs-loaded CEVs can increase release of IL-2 and IFN-γ and reduce of CD163+ macrophages in malignant pleural effusion (25). Nevertheless, the side effect of transient decrease in the absolute numbers of lymphoid cells could be observed.

However, the low production of CEVs remains a challenge for clinical application. Compared with the CEVs from MPE, the autologous tumor cell-derived extracellular vesicles are not always available in patients with solid tumors for the preparation of CEVs. There is an urgent need for the development of an innovative strategy for the large-scale production of CEVs.

## Conclusions

Despite the predetermined notions regarding the role of CEVs based on their contents, the therapeutic role of phospholipid bilayer membrane-coated EVs prepared from cancer cells was explored. The CEVs have shown excellent potential as anti-cancer agents. The strategies employ diverse generation, application, and therapeutic mechanisms. The main strategies for CEVs generation have been summarized in four categories, including irradiated CEVs, advanced materials-combined CEVs, chemotherapeutic drug-loaded CEVs, and genetically engineered CEVs (**Table 1**). Along with the increasing challenges and advantages of CEVs for cancer therapy, the exploration of CEVs is becoming more and more necessary. This review also discussed and categorized the future perspectives and the top three challenges for the preparation, efficacy, and application of CEVs.

**Table 1:** The summative evaluation for the preparation, therapeutic mechanism and challenges of cancer cells-derived extracellular vesicles.

Classification	Source of CEVs	Preparation of specific CEVs		Anti-tumor Effects	Model	Challenges
		Key strategies	Purification strategies			
Irradiated CEVs	Spontaneous release from LLC (35) MCF-7, A549 (25, 52) CT26,EG7 (49), HCT116 (50)	Ultraviolet (UVB, 300J m-2) irradiation for l hour (25, 52) 20Gy by 6-MV x-rays(600 MU/min) (35) 50 or 75 Gy X-ray irradiation (49) Irradiated with 2x2 Gy or 2x5 Gy (51)	Sequential utracentrifugation (25, 35) (at least 100,000 g for 60 min) Filtration With 0.22m filter combined sequential utracentrifugation (51) Sequential centrifugation (52) (up to 14,000 g for 60 min)	The induced Ferroptosis of cancer cells (35) Activation of dendritic cells and T cells (49–51) The reduced resistance of cisplatin (31) Polarizes microenvironmental M2 tumor-associated macrophages (M2-TAMs)to M1-TAMs (35, 51)	Mouse model of malignant pleural effusion (25, 35) Mice model bearing subcutaneous tumor (49–51) Clinical experiment in patients with lung cancer (52)	Preparation: Limited Volume of CEV Expensive and complecated protocol of collection Storage: Unstable construction and targetability during preparation and preservation
Advanced materials combined CEVs	Membranes from the lysis of cancer cells(A5S9,B16-F10) (58) KBcells (59), MCF-7 (18),MDA-MB-435 (63) and hybrid cells of 4Tl with dendritic cells (16)	CEVs coated with anionic PLGA microspheres via click chemistry (58) The mixture of ZnO and CEVs was filtered with 0.2μm filter followed by centrifugation at 5000 g for 5 min (59) Co-extrusion of the mixture of photothermal materials loaded-nanoparticles and membrane to generate biomimetic CEVs (16, 18, 63)	Removal of unreacted particles and chemicals via 8000 rpm for 10 min (58) Repeated coincubation,filteration and centrifugation with ZnO (59) Repeated the process of co-extrusion through a filter with 220 or 400 nm polycarbonate membrane (16, 18, 63)	Increased phagocytosis of macrophages and dendritic cells (58) Direct killing effect of ZnO (59) Tumor-targeted CEVs combined photothermal effect on tumor area (16, 18, 63) Increased recruitment and activation of T cells (16)	*In vitro* experiment (58, 59) Mice model bearing subcutaneous tumoR (16, 18, 63)	Administration: The clearance of CEVs in serum for intravenous administration The loss of CEVs in organs for intravenous administration
Chemotherapeutic drugs loaded CEVs	Spontaneous release from drug-engulfed cancer cells (21–23, 25, 51) Spontaneous release from wild cancer cells (19, 65–67)	The engulfing process of drugs is conducted via the incubation of cancers cells with drugs so as to release drug-loaded CEVs (21–23, 25, 51) Co-extrusion of the mixture of CEVs and drugs was performed after the centifugation and incubation of the mixture at 4^o^C (19) The CEVs and drugs were incubated in saponin at 37^o^C (65) the Freeze-thaw cycles was performed after the stirring process of CEVs and drugs (62) The CEVs were incubated with drugs at 22^o^C or 37^o^C in the solution of drugs (67)	The drugs-loaded CEVs containing supernatants were received a and repeated and sequential utracentrifugation (21–23, 25, 51) The excess drugs was removed by filtering through an Amicon Ultra-15 100 kDa filter (19) The extral drugs and saponin were remove with Nanosep device (65) The mixtures of drugs-CEVs were filter through protein concentrators(MicroSep Centrifugal Devices 10k) at 2,000x g for 10 min (62) Repeated utracentrifugation (67) (up to 170,000 g for 120 min)	The direct killing effect via tumor-targeted CEVs combined chemotherapeutic drugs (19, 21–23, 25, 51, 65–67) Increased brogation of tumor-repopulating cells (18) The increased reactive oxygen species (ROS)and neutrophil extracellular trap (NET) in tumor area (18) The increaed release of IL-2 and IFN-_Ɣ_ and reduce of CDI67+ macrophagesin malignant pleual effusion (25)	*In vitro* txptriment (65–67) Mice model bearing subcutaneous or internal tumor (19, 21–23, 51) Mice model bearing malignant pleural effusion (25) Clinicalt experiment in patients with malignant pleural effusion (25)	Side Effects: Natural CEVs are promotors to tumor progression,tumor metastasis Exogenous CEVs can be allergen for recipient Transient decrease in the absolute numbers of lymphoid cells
Genetically Engineered CEVs	Spontaneous release from A549 (28) 4Tl cells (30) Panc-l (31) BL6BL6 cells (33)	Rab3la overexpression vector was transfected into A549 (28) The plasmid vectors expressing anti-miR-21 is directly transfected to 4T1 cells (30) Panc-l was transfected with plasmid DNA complexed with Lipofectamine® 3000 (31) Aplasmid vector encoding a streptavidin-lactadherin fusion protein was transfected with BL6BL6 cells (33)	Sequentially repeated ultracentrifugation (28, 30, 33) (up to 250,000g/100,000g for 120 min) Precipitation Solution (31)	Induced maturation of dendritic cells and promoted prolification of CD4+ T cell (28) Attenuated resistance of doxorubicin(DOX) (30) The promotion of reprogramming of macrophages to the M1 phenotype (31) Enhanced tumor antigen presentation capacity by MHC class 1 molecules (33)	*In vitro* experiment (31, 33) Mice model bearing subcutaneous tumor (28, 30)	Application: Limited homologous CEVs in clinical The efficacy of CEVs differs FROM TUMORS Limited standard for the dose of CEVs

## Author Contributions

WL was in charge of all the writing and graphics. X-DC was in charge of the design and instruction. All authors contributed to the article and approved the submitted version.

## Conflict of Interest

The authors declare that the research was conducted in the absence of any commercial or financial relationships that could be construed as a potential conflict of interest.

## Publisher’s Note

All claims expressed in this article are solely those of the authors and do not necessarily represent those of their affiliated organizations, or those of the publisher, the editors and the reviewers. Any product that may be evaluated in this article, or claim that may be made by its manufacturer, is not guaranteed or endorsed by the publisher.
